# Triethyl­ammonium 2,4,6-trisulfanylidene-1,3,5-triazinan-1-ide

**DOI:** 10.1107/S1600536811034271

**Published:** 2011-08-27

**Authors:** He-Ping Li, Seik Weng Ng

**Affiliations:** a Henan University of Traditional Chinese Medicine, Zhengzhou 450008, People’s Republic of China; b Department of Chemistry, University of Malaya, 50603 Kuala Lumpur, Malaysia; c Chemistry Department, King Abdulaziz University, PO Box 80203, Jeddah, Saudi Arabia

## Abstract

The asymmetric unit of the title compound, C_6_H_16_N^+^·C_3_H_2_N_3_S_3_^−^, contains two independent ion pairs. The 2,4,6-trithioxo-1,3,5-triazinan-1-ide anion features an almost planar six-membered ring (r.m.s. deviations = 0.009 and 0.018 Å) having exocyclic double-bond S atoms. The anions inter­act by N—H⋯S hydrogen bonds to generate a chain running along [110]. The triethyl­ammonium cations are hydrogen bonded to the anions with the ammonium H atom forming a hydrogen bond to the negatively-charged N atom of the anion. In the crystal structure, both triethyl­ammonium cations are disordered over two orientations with equal occupancies.

## Related literature

For trimethyl­ammonium 2,4,6-trithioxo-1,3,5-triazinan-1-ide monohydrate, see: Hou & Yang (2011 ▶).
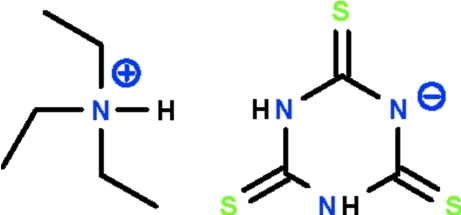


## Experimental

### 

#### Crystal data

C_6_H_16_N^+^·C_3_H_2_N_3_S_3_^−^
*M* *_r_* = 278.45
Monoclinic, 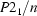

*a* = 13.1648 (3) Å
*b* = 13.0636 (3) Å
*c* = 16.9552 (4) Å
β = 93.779 (2)°
*V* = 2909.61 (12) Å^3^
*Z* = 8
Mo *K*α radiation
μ = 0.49 mm^−1^
*T* = 293 K
0.40 × 0.30 × 0.20 mm

#### Data collection

Bruker SMART APEX diffractometer
Absorption correction: multi-scan (*SADABS*; Sheldrick, 1996 ▶) *T*_min_ = 0.828, *T*_max_ = 0.908
13126 measured reflections
5978 independent reflections
3218 reflections with *I* > 2σ(*I*)
*R*_int_ = 0.026

#### Refinement

*R*[*F* ^2^ > 2σ(*F* ^2^)] = 0.060
*wR*(*F* ^2^) = 0.196
*S* = 1.02
5978 reflections
347 parameters
136 restraints
H atoms treated by a mixture of independent and constrained refinement
Δρ_max_ = 0.41 e Å^−3^
Δρ_min_ = −0.29 e Å^−3^

### 

Data collection: *APEX2* (Bruker, 2007 ▶); cell refinement: *SAINT* (Bruker, 2007 ▶); data reduction: *SAINT*; program(s) used to solve structure: *SHELXS97* (Sheldrick, 2008 ▶); program(s) used to refine structure: *SHELXL97* (Sheldrick, 2008 ▶); molecular graphics: *X-SEED* (Barbour, 2001 ▶); software used to prepare material for publication: *publCIF* (Westrip, 2010 ▶).

## Supplementary Material

Crystal structure: contains datablock(s) global, I. DOI: 10.1107/S1600536811034271/xu5300sup1.cif

Supplementary material file. DOI: 10.1107/S1600536811034271/xu5300Isup3.cml

Structure factors: contains datablock(s) I. DOI: 10.1107/S1600536811034271/xu5300Isup2.hkl

Additional supplementary materials:  crystallographic information; 3D view; checkCIF report

## Figures and Tables

**Table 1:** Hydrogen-bond geometry (Å, °)

*D*—H⋯*A*	*D*—H	H⋯*A*	*D*⋯*A*	*D*—H⋯*A*
N1—H1⋯N4	0.88	2.03	2.89 (1)	163
N1′—H1′⋯N4	0.88	2.08	2.95 (1)	166
N2—H2⋯N7	0.88	2.02	2.88 (1)	165
N2′—H2′⋯N7	0.88	2.04	2.89 (1)	163
N3—H3⋯S4	0.88 (1)	2.38 (1)	3.248 (3)	169 (4)
N5—H5⋯S2^i^	0.88 (1)	2.44 (1)	3.319 (4)	171 (3)
N6—H6⋯S1	0.87 (1)	2.58 (1)	3.446 (3)	170 (3)
N8—H8⋯S6^ii^	0.88 (1)	2.47 (2)	3.326 (3)	164 (4)

Symmetry codes: (i) 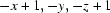; (ii) 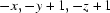.

**Fig. 1.**
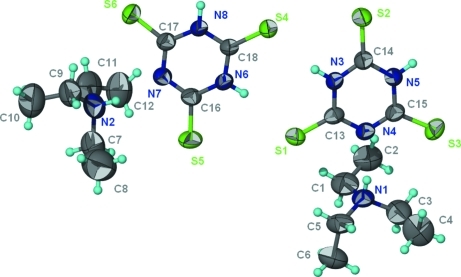
Thermal ellipsoid plot (Barbour, 2001) of the two independent ion-pairs of C6H16N.C3H2N3S2 at the 50% probability level; hydrogen atoms are drawn as spheres of arbitrary radius. The disorder in the cations is not shown.

## supplementary crystallographic information

### Comment

This study is an extension of a study on the amine salts of 1,3,5-triazinane-2,4,6-trithione (thiocyanuric acid). The previous study described the monohydrated trimethylammonium salt (Hou & Yang, 2011). The triethylammonium analog (Scheme I) does not crystallize with water. Its anion features a planar six-membered ring having exocyclic double-bond S atoms (Fig. 1). The anions interact by N–H···S hydrogen bonds to generate a chain running along [1 1 0] (Table 1). The cations are hydrogen bonded to the anions with the ammonium H forming a hydrogen bond to the negatively-charged N atom of the anion.

### Experimental

1,3,5-Triazin-2,4,6-trithione (0.25 mmol, 0.045 g) was dissolved in a water-ethanol (50/100 *v*/*v*) mixture. Triethylamine (0.75 mmol, 0.076 g) was added to the solution. The mixture was stirred and then set aside for the growth of colorless crystals, which appeared after several weeks.

### Refinement

Carbon-bound H-atoms were placed in calculated positions (C—H 0.95 to 0.98 Å) and were included in the refinement in the riding model approximation, with *U*(H) set to 1.2 to 1.5*U*(C).

Both triethylammonium cations are disordered over two positions; the disorder could not be refined, and was assumed to be a 1:1 type of disorder. The 1,2-connected C–C and C–N distances were restrained to 1.50±0.01 Å and the 1,3-related non-bonded ones to 2.51±0.01 Å. The temperature factors of the primed atoms were set to those of the unprimed ones, and the anisotropic temperature factors were restrained to be nearly isotropic.

The nitrogen-bound H-atoms of the cation were treated in the riding model approximation; those of the anion were located in a difference Fourier map, and were refined with a distance restraint of N–H 0.884±0.01 Å; their temperature factors were freely refined.

### Crystal data

**Table:**

C_6_H_16_N^+^·C_3_H_2_N_3_S_3_^−^	*F*(000) = 1184
*M**_r_* = 278.45	*D*_x_ = 1.271 Mg m^−^^3^
Monoclinic, *P*2_1_/*n*	Mo *K*α radiation, λ = 0.71073 Å
Hall symbol: -P 2yn	Cell parameters from 2936 reflections
*a* = 13.1648 (3) Å	θ = 2.5–24.1°
*b* = 13.0636 (3) Å	µ = 0.49 mm^−^^1^
*c* = 16.9552 (4) Å	*T* = 293 K
β = 93.779 (2)°	Prism, colorless
*V* = 2909.61 (12) Å^3^	0.40 × 0.30 × 0.20 mm
*Z* = 8	

### Data collection

**Table:**

Bruker SMART APEX diffractometer	5978 independent reflections
Radiation source: fine-focus sealed tube	3218 reflections with *I* > 2σ(*I*)
graphite	*R*_int_ = 0.026
ω scans	θ_max_ = 26.5°, θ_min_ = 2.0°
Absorption correction: multi-scan (*SADABS*; Sheldrick, 1996)	*h* = −16→15
*T*_min_ = 0.828, *T*_max_ = 0.908	*k* = −13→16
13126 measured reflections	*l* = −21→21

### Refinement

**Table:**

Refinement on *F*^2^	Primary atom site location: structure-invariant direct methods
Least-squares matrix: full	Secondary atom site location: difference Fourier map
*R*[*F*^2^ > 2σ(*F*^2^)] = 0.060	Hydrogen site location: inferred from neighbouring sites
*wR*(*F*^2^) = 0.196	H atoms treated by a mixture of independent and constrained refinement
*S* = 1.02	*w* = 1/[σ^2^(*F*_o_^2^) + (0.0954*P*)^2^ + 0.927*P*] where *P* = (*F*_o_^2^ + 2*F*_c_^2^)/3
5978 reflections	(Δ/σ)_max_ = 0.001
347 parameters	Δρ_max_ = 0.41 e Å^−^^3^
136 restraints	Δρ_min_ = −0.29 e Å^−^^3^

### Fractional atomic coordinates and isotropic or equivalent isotropic displacement parameters (Å^2^)

**Table:**

	*x*	*y*	*z*	*U*_iso_*/*U*_eq_	Occ. (<1)
S1	0.56457 (8)	0.44837 (8)	0.61142 (7)	0.0707 (3)	
S2	0.39743 (9)	0.12143 (9)	0.47969 (8)	0.0934 (5)	
S3	0.77911 (9)	0.10693 (11)	0.59146 (9)	0.0958 (5)	
S4	0.26844 (8)	0.35815 (9)	0.50491 (8)	0.0850 (4)	
S5	0.39625 (9)	0.69261 (12)	0.65071 (9)	0.1040 (5)	
S6	0.00526 (8)	0.63977 (11)	0.56829 (9)	0.1017 (5)	
N1	0.8202 (8)	0.3621 (7)	0.7084 (6)	0.076 (2)	0.50
H1	0.7845	0.3346	0.6682	0.091*	0.50
N1'	0.8362 (8)	0.3333 (8)	0.7120 (6)	0.076 (2)	0.50
H1'	0.7947	0.3129	0.6722	0.091*	0.50
N2	0.1569 (6)	0.8543 (8)	0.6796 (6)	0.1009 (14)	0.50
H2	0.1816	0.8015	0.6550	0.121*	0.50
N2'	0.1547 (6)	0.8603 (8)	0.6708 (6)	0.1009 (14)	0.50
H2'	0.1577	0.8023	0.6447	0.121*	0.50
N3	0.5004 (2)	0.2787 (3)	0.54226 (18)	0.0621 (8)	
N4	0.6686 (2)	0.2755 (3)	0.59703 (17)	0.0624 (8)	
N5	0.5888 (2)	0.1310 (3)	0.5391 (2)	0.0669 (9)	
N6	0.3181 (2)	0.5339 (3)	0.57618 (18)	0.0604 (8)	
N7	0.2027 (2)	0.6633 (3)	0.60457 (18)	0.0640 (8)	
N8	0.1518 (2)	0.5117 (2)	0.53993 (19)	0.0620 (8)	
C1	0.7544 (7)	0.3568 (7)	0.7780 (5)	0.108 (3)	0.50
H1A	0.7888	0.3916	0.8228	0.129*	0.50
H1B	0.6909	0.3924	0.7648	0.129*	0.50
C2	0.7317 (11)	0.2483 (8)	0.8011 (8)	0.121 (4)	0.50
H2A	0.6885	0.2486	0.8445	0.182*	0.50
H2B	0.7942	0.2137	0.8165	0.182*	0.50
H2C	0.6981	0.2134	0.7569	0.182*	0.50
C3	0.9125 (11)	0.2955 (13)	0.7237 (8)	0.122 (3)	0.50
H3A	0.9463	0.3149	0.7740	0.147*	0.50
H3B	0.8904	0.2250	0.7281	0.147*	0.50
C4	0.9878 (11)	0.3016 (15)	0.6613 (7)	0.142 (4)	0.50
H4A	1.0450	0.2583	0.6757	0.213*	0.50
H4B	1.0106	0.3710	0.6566	0.213*	0.50
H4C	0.9561	0.2795	0.6116	0.213*	0.50
C5	0.8420 (11)	0.4699 (8)	0.6851 (7)	0.097 (3)	0.50
H5A	0.7780	0.5064	0.6769	0.117*	0.50
H5B	0.8739	0.4689	0.6351	0.117*	0.50
C6	0.9096 (18)	0.5278 (12)	0.7443 (11)	0.124 (5)	0.50
H6A	0.9188	0.5965	0.7258	0.186*	0.50
H6B	0.9746	0.4944	0.7509	0.186*	0.50
H6C	0.8788	0.5296	0.7940	0.186*	0.50
C7	0.2470 (8)	0.9181 (9)	0.7086 (6)	0.136 (3)	0.50
H7A	0.2235	0.9747	0.7397	0.163*	0.50
H7B	0.2923	0.8766	0.7428	0.163*	0.50
C8	0.3050 (12)	0.9597 (14)	0.6424 (10)	0.203 (6)	0.50
H8A	0.3612	1.0000	0.6638	0.305*	0.50
H8B	0.2608	1.0018	0.6087	0.305*	0.50
H8C	0.3300	0.9040	0.6122	0.305*	0.50
C9	0.0883 (8)	0.9094 (9)	0.6200 (5)	0.119 (3)	0.50
H9A	0.0346	0.8634	0.6005	0.143*	0.50
H9B	0.1270	0.9289	0.5756	0.143*	0.50
C10	0.0415 (12)	1.0034 (10)	0.6533 (10)	0.181 (5)	0.50
H10A	−0.0038	1.0345	0.6136	0.271*	0.50
H10B	0.0942	1.0511	0.6698	0.271*	0.50
H10C	0.0042	0.9848	0.6979	0.271*	0.50
C11	0.0997 (8)	0.8114 (10)	0.7461 (7)	0.143 (4)	0.50
H11A	0.0848	0.8665	0.7819	0.171*	0.50
H11B	0.0354	0.7836	0.7246	0.171*	0.50
C12	0.1574 (12)	0.7292 (13)	0.7917 (10)	0.156 (5)	0.50
H12A	0.1333	0.7246	0.8438	0.234*	0.50
H12B	0.2286	0.7457	0.7955	0.234*	0.50
H12C	0.1471	0.6649	0.7651	0.234*	0.50
C1'	0.8025 (7)	0.2833 (8)	0.7858 (5)	0.108 (3)	0.50
H1'A	0.8058	0.2097	0.7790	0.129*	0.50
H1'B	0.8507	0.3013	0.8293	0.129*	0.50
C2'	0.6989 (8)	0.3099 (11)	0.8085 (8)	0.121 (4)	0.50
H2'A	0.6834	0.2709	0.8541	0.182*	0.50
H2'B	0.6502	0.2944	0.7655	0.182*	0.50
H2'C	0.6960	0.3816	0.8205	0.182*	0.50
C3'	0.9402 (10)	0.2901 (14)	0.6995 (8)	0.122 (3)	0.50
H3'A	0.9880	0.3147	0.7411	0.147*	0.50
H3'B	0.9375	0.2161	0.7033	0.147*	0.50
C4'	0.9777 (12)	0.3186 (15)	0.6218 (7)	0.142 (4)	0.50
H4'A	1.0491	0.3037	0.6218	0.213*	0.50
H4'B	0.9668	0.3903	0.6126	0.213*	0.50
H4'C	0.9415	0.2800	0.5807	0.213*	0.50
C5'	0.8326 (11)	0.4481 (8)	0.7142 (8)	0.097 (3)	0.50
H5'A	0.7668	0.4692	0.7309	0.117*	0.50
H5'B	0.8388	0.4741	0.6611	0.117*	0.50
C6'	0.9144 (17)	0.4957 (12)	0.7684 (12)	0.124 (5)	0.50
H6'A	0.9082	0.5688	0.7663	0.186*	0.50
H6'B	0.9800	0.4759	0.7521	0.186*	0.50
H6'C	0.9072	0.4726	0.8216	0.186*	0.50
C7'	0.2308 (8)	0.9288 (8)	0.6358 (7)	0.136 (3)	0.50
H7'A	0.2105	0.9400	0.5805	0.163*	0.50
H7'B	0.2965	0.8949	0.6387	0.163*	0.50
C8'	0.2415 (14)	1.0306 (9)	0.6767 (11)	0.203 (6)	0.50
H8'A	0.2899	1.0718	0.6510	0.305*	0.50
H8'B	0.2647	1.0203	0.7310	0.305*	0.50
H8'C	0.1767	1.0646	0.6741	0.305*	0.50
C9'	0.0469 (7)	0.8951 (10)	0.6579 (6)	0.119 (3)	0.50
H9'A	0.0363	0.9524	0.6929	0.143*	0.50
H9'B	0.0025	0.8400	0.6726	0.143*	0.50
C10'	0.0164 (11)	0.9271 (14)	0.5749 (7)	0.181 (5)	0.50
H10D	−0.0533	0.9490	0.5716	0.271*	0.50
H10E	0.0241	0.8703	0.5399	0.271*	0.50
H10F	0.0590	0.9825	0.5600	0.271*	0.50
C11'	0.1796 (9)	0.8327 (10)	0.7566 (6)	0.143 (4)	0.50
H11C	0.2496	0.8095	0.7636	0.171*	0.50
H11D	0.1723	0.8927	0.7895	0.171*	0.50
C12'	0.1092 (12)	0.7493 (13)	0.7816 (10)	0.156 (5)	0.50
H12D	0.1256	0.7323	0.8360	0.234*	0.50
H12E	0.1172	0.6897	0.7494	0.234*	0.50
H12F	0.0400	0.7727	0.7753	0.234*	0.50
C13	0.5803 (3)	0.3276 (3)	0.5823 (2)	0.0592 (9)	
C14	0.5002 (3)	0.1797 (3)	0.5217 (2)	0.0636 (10)	
C15	0.6742 (3)	0.1784 (3)	0.5752 (2)	0.0640 (10)	
C16	0.2983 (3)	0.6290 (3)	0.6081 (2)	0.0633 (10)	
C17	0.1280 (3)	0.6037 (3)	0.5719 (2)	0.0641 (10)	
C18	0.2463 (3)	0.4720 (3)	0.5414 (2)	0.0573 (9)	
H3	0.4412 (16)	0.309 (3)	0.532 (2)	0.075 (12)*	
H5	0.589 (3)	0.0647 (10)	0.529 (2)	0.058 (11)*	
H6	0.3782 (15)	0.506 (3)	0.581 (2)	0.071 (12)*	
H8	0.101 (2)	0.475 (3)	0.519 (2)	0.093 (14)*	

### Atomic displacement parameters (Å^2^)

**Table:**

	*U*^11^	*U*^22^	*U*^33^	*U*^12^	*U*^13^	*U*^23^
S1	0.0648 (6)	0.0647 (7)	0.0806 (7)	−0.0049 (5)	−0.0096 (5)	−0.0097 (6)
S2	0.0712 (7)	0.0719 (8)	0.1298 (11)	0.0086 (5)	−0.0474 (7)	−0.0257 (7)
S3	0.0604 (7)	0.1016 (10)	0.1215 (10)	0.0198 (6)	−0.0237 (6)	−0.0220 (8)
S4	0.0596 (6)	0.0703 (8)	0.1218 (10)	0.0075 (5)	−0.0196 (6)	−0.0299 (7)
S5	0.0692 (7)	0.1147 (11)	0.1265 (11)	−0.0177 (7)	−0.0060 (7)	−0.0590 (9)
S6	0.0572 (6)	0.1002 (10)	0.1459 (12)	0.0125 (6)	−0.0065 (7)	−0.0554 (9)
N1	0.066 (3)	0.081 (5)	0.077 (2)	−0.008 (3)	−0.023 (2)	−0.001 (3)
N1'	0.066 (3)	0.081 (5)	0.077 (2)	−0.008 (3)	−0.023 (2)	−0.001 (3)
N2	0.115 (3)	0.091 (3)	0.099 (3)	0.003 (3)	0.028 (3)	−0.028 (3)
N2'	0.115 (3)	0.091 (3)	0.099 (3)	0.003 (3)	0.028 (3)	−0.028 (3)
N3	0.0509 (18)	0.063 (2)	0.071 (2)	0.0060 (15)	−0.0113 (15)	−0.0105 (17)
N4	0.0521 (17)	0.071 (2)	0.0620 (18)	0.0003 (15)	−0.0100 (13)	−0.0100 (17)
N5	0.0577 (19)	0.061 (2)	0.079 (2)	0.0062 (16)	−0.0188 (16)	−0.0142 (18)
N6	0.0460 (17)	0.070 (2)	0.0646 (19)	0.0004 (15)	−0.0022 (14)	−0.0133 (17)
N7	0.0582 (18)	0.070 (2)	0.0633 (19)	−0.0020 (16)	0.0029 (14)	−0.0212 (17)
N8	0.0449 (17)	0.062 (2)	0.078 (2)	−0.0008 (15)	−0.0038 (15)	−0.0179 (18)
C1	0.107 (6)	0.122 (7)	0.089 (4)	−0.013 (5)	−0.027 (4)	0.003 (5)
C2	0.120 (7)	0.143 (9)	0.102 (5)	−0.014 (6)	0.013 (5)	0.005 (7)
C3	0.117 (7)	0.106 (5)	0.137 (8)	0.007 (5)	−0.041 (5)	−0.003 (6)
C4	0.185 (7)	0.155 (7)	0.094 (8)	−0.018 (5)	0.071 (7)	0.015 (7)
C5	0.086 (4)	0.096 (6)	0.107 (8)	−0.026 (4)	−0.011 (5)	0.031 (5)
C6	0.125 (5)	0.114 (8)	0.130 (9)	−0.035 (6)	−0.001 (6)	−0.013 (7)
C7	0.142 (6)	0.122 (6)	0.146 (7)	0.014 (5)	0.018 (7)	−0.042 (7)
C8	0.203 (9)	0.183 (9)	0.225 (9)	−0.031 (8)	0.034 (8)	−0.022 (8)
C9	0.122 (7)	0.108 (5)	0.127 (7)	0.018 (6)	0.005 (5)	−0.028 (6)
C10	0.168 (8)	0.164 (8)	0.210 (9)	0.045 (7)	0.002 (7)	−0.015 (7)
C11	0.150 (8)	0.158 (7)	0.125 (6)	0.013 (7)	0.049 (7)	−0.044 (6)
C12	0.182 (10)	0.164 (8)	0.126 (6)	0.042 (8)	0.043 (8)	−0.010 (6)
C1'	0.107 (6)	0.122 (7)	0.089 (4)	−0.013 (5)	−0.027 (4)	0.003 (5)
C2'	0.120 (7)	0.143 (9)	0.102 (5)	−0.014 (6)	0.013 (5)	0.005 (7)
C3'	0.117 (7)	0.106 (5)	0.137 (8)	0.007 (5)	−0.041 (5)	−0.003 (6)
C4'	0.185 (7)	0.155 (7)	0.094 (8)	−0.018 (5)	0.071 (7)	0.015 (7)
C5'	0.086 (4)	0.096 (6)	0.107 (8)	−0.026 (4)	−0.011 (5)	0.031 (5)
C6'	0.125 (5)	0.114 (8)	0.130 (9)	−0.035 (6)	−0.001 (6)	−0.013 (7)
C7'	0.142 (6)	0.122 (6)	0.146 (7)	0.014 (5)	0.018 (7)	−0.042 (7)
C8'	0.203 (9)	0.183 (9)	0.225 (9)	−0.031 (8)	0.034 (8)	−0.022 (8)
C9'	0.122 (7)	0.108 (5)	0.127 (7)	0.018 (6)	0.005 (5)	−0.028 (6)
C10'	0.168 (8)	0.164 (8)	0.210 (9)	0.045 (7)	0.002 (7)	−0.015 (7)
C11'	0.150 (8)	0.158 (7)	0.125 (6)	0.013 (7)	0.049 (7)	−0.044 (6)
C12'	0.182 (10)	0.164 (8)	0.126 (6)	0.042 (8)	0.043 (8)	−0.010 (6)
C13	0.051 (2)	0.075 (3)	0.051 (2)	−0.0053 (18)	−0.0031 (15)	−0.0016 (19)
C14	0.060 (2)	0.067 (3)	0.061 (2)	0.0059 (18)	−0.0141 (17)	−0.007 (2)
C15	0.052 (2)	0.078 (3)	0.060 (2)	0.0025 (18)	−0.0090 (16)	−0.009 (2)
C16	0.062 (2)	0.074 (3)	0.054 (2)	−0.0113 (19)	0.0054 (17)	−0.016 (2)
C17	0.057 (2)	0.070 (3)	0.064 (2)	−0.0033 (19)	0.0018 (18)	−0.013 (2)
C18	0.052 (2)	0.060 (2)	0.059 (2)	−0.0046 (17)	−0.0047 (16)	−0.0050 (18)

### Geometric parameters (Å, °)

**Table:**

S1—C13	1.669 (4)	C6—H6C	0.9600
S2—C14	1.670 (4)	C7—C8	1.501 (5)
S3—C15	1.675 (4)	C7—H7A	0.9700
S4—C18	1.645 (4)	C7—H7B	0.9700
S5—C16	1.659 (4)	C8—H8A	0.9600
S6—C17	1.681 (4)	C8—H8B	0.9600
N1—C5	1.496 (5)	C8—H8C	0.9600
N1—C3	1.502 (5)	C9—C10	1.501 (5)
N1—C1	1.511 (5)	C9—H9A	0.9700
N1—H1	0.8800	C9—H9B	0.9700
N1'—C5'	1.501 (5)	C10—H10A	0.9600
N1'—C1'	1.505 (5)	C10—H10B	0.9600
N1'—C3'	1.508 (5)	C10—H10C	0.9600
N1'—H1'	0.8800	C11—C12	1.499 (5)
N2—C9	1.495 (5)	C11—H11A	0.9700
N2—C11	1.505 (5)	C11—H11B	0.9700
N2—C7	1.505 (5)	C12—H12A	0.9600
N2—H2	0.8800	C12—H12B	0.9600
N2'—C9'	1.493 (5)	C12—H12C	0.9600
N2'—C7'	1.494 (5)	C1'—C2'	1.482 (5)
N2'—C11'	1.514 (5)	C1'—H1'A	0.9700
N2'—H2'	0.8800	C1'—H1'B	0.9700
N3—C14	1.339 (5)	C2'—H2'A	0.9600
N3—C13	1.373 (4)	C2'—H2'B	0.9600
N3—H3	0.881 (10)	C2'—H2'C	0.9600
N4—C15	1.325 (5)	C3'—C4'	1.484 (5)
N4—C13	1.356 (4)	C3'—H3'A	0.9700
N5—C14	1.345 (4)	C3'—H3'B	0.9700
N5—C15	1.389 (4)	C4'—H4'A	0.9600
N5—H5	0.883 (10)	C4'—H4'B	0.9600
N6—C18	1.350 (4)	C4'—H4'C	0.9600
N6—C16	1.387 (5)	C5'—C6'	1.504 (5)
N6—H6	0.873 (10)	C5'—H5'A	0.9700
N7—C16	1.333 (5)	C5'—H5'B	0.9700
N7—C17	1.345 (5)	C6'—H6'A	0.9600
N8—C18	1.347 (5)	C6'—H6'B	0.9600
N8—C17	1.362 (5)	C6'—H6'C	0.9600
N8—H8	0.881 (10)	C7'—C8'	1.501 (5)
C1—C2	1.506 (5)	C7'—H7'A	0.9700
C1—H1A	0.9700	C7'—H7'B	0.9700
C1—H1B	0.9700	C8'—H8'A	0.9600
C2—H2A	0.9600	C8'—H8'B	0.9600
C2—H2B	0.9600	C8'—H8'C	0.9600
C2—H2C	0.9600	C9'—C10'	1.498 (5)
C3—C4	1.500 (5)	C9'—H9'A	0.9700
C3—H3A	0.9700	C9'—H9'B	0.9700
C3—H3B	0.9700	C10'—H10D	0.9600
C4—H4A	0.9600	C10'—H10E	0.9600
C4—H4B	0.9600	C10'—H10F	0.9600
C4—H4C	0.9600	C11'—C12'	1.510 (5)
C5—C6	1.502 (5)	C11'—H11C	0.9700
C5—H5A	0.9700	C11'—H11D	0.9700
C5—H5B	0.9700	C12'—H12D	0.9600
C6—H6A	0.9600	C12'—H12E	0.9600
C6—H6B	0.9600	C12'—H12F	0.9600
			
C5—N1—C3	115.1 (6)	N2—C11—H11A	109.0
C5—N1—C1	112.3 (6)	C12—C11—H11B	109.0
C3—N1—C1	109.7 (6)	N2—C11—H11B	109.0
C5—N1—H1	106.4	H11A—C11—H11B	107.8
C3—N1—H1	106.4	C11—C12—H12A	109.5
C1—N1—H1	106.4	C11—C12—H12B	109.5
C5'—N1'—C1'	113.7 (6)	H12A—C12—H12B	109.5
C5'—N1'—C3'	114.1 (7)	C11—C12—H12C	109.5
C1'—N1'—C3'	106.0 (5)	H12A—C12—H12C	109.5
C5'—N1'—H1'	107.6	H12B—C12—H12C	109.5
C1'—N1'—H1'	107.6	C2'—C1'—N1'	116.2 (7)
C3'—N1'—H1'	107.6	C2'—C1'—H1'A	108.2
C9—N2—C11	112.1 (6)	N1'—C1'—H1'A	108.2
C9—N2—C7	112.5 (6)	C2'—C1'—H1'B	108.2
C11—N2—C7	112.6 (7)	N1'—C1'—H1'B	108.2
C9—N2—H2	106.4	H1'A—C1'—H1'B	107.4
C11—N2—H2	106.4	C1'—C2'—H2'A	109.5
C7—N2—H2	106.4	C1'—C2'—H2'B	109.5
C9'—N2'—C7'	114.6 (6)	H2'A—C2'—H2'B	109.5
C9'—N2'—C11'	110.9 (6)	C1'—C2'—H2'C	109.5
C7'—N2'—C11'	114.6 (6)	H2'A—C2'—H2'C	109.5
C9'—N2'—H2'	105.3	H2'B—C2'—H2'C	109.5
C7'—N2'—H2'	105.3	C4'—C3'—N1'	112.9 (7)
C11'—N2'—H2'	105.3	C4'—C3'—H3'A	109.0
C14—N3—C13	124.6 (3)	N1'—C3'—H3'A	109.0
C14—N3—H3	113 (3)	C4'—C3'—H3'B	109.0
C13—N3—H3	122 (3)	N1'—C3'—H3'B	109.0
C15—N4—C13	119.5 (3)	H3'A—C3'—H3'B	107.8
C14—N5—C15	123.4 (4)	C3'—C4'—H4'A	109.5
C14—N5—H5	115 (2)	C3'—C4'—H4'B	109.5
C15—N5—H5	121 (2)	H4'A—C4'—H4'B	109.5
C18—N6—C16	124.2 (3)	C3'—C4'—H4'C	109.5
C18—N6—H6	113 (3)	H4'A—C4'—H4'C	109.5
C16—N6—H6	123 (3)	H4'B—C4'—H4'C	109.5
C16—N7—C17	119.1 (3)	N1'—C5'—C6'	113.9 (7)
C18—N8—C17	124.6 (3)	N1'—C5'—H5'A	108.8
C18—N8—H8	118 (3)	C6'—C5'—H5'A	108.8
C17—N8—H8	117 (3)	N1'—C5'—H5'B	108.8
C2—C1—N1	112.3 (6)	C6'—C5'—H5'B	108.8
C2—C1—H1A	109.1	H5'A—C5'—H5'B	107.7
N1—C1—H1A	109.1	C5'—C6'—H6'A	109.5
C2—C1—H1B	109.1	C5'—C6'—H6'B	109.5
N1—C1—H1B	109.1	H6'A—C6'—H6'B	109.5
H1A—C1—H1B	107.9	C5'—C6'—H6'C	109.5
C1—C2—H2A	109.5	H6'A—C6'—H6'C	109.5
C1—C2—H2B	109.5	H6'B—C6'—H6'C	109.5
H2A—C2—H2B	109.5	N2'—C7'—C8'	113.1 (7)
C1—C2—H2C	109.5	N2'—C7'—H7'A	109.0
H2A—C2—H2C	109.5	C8'—C7'—H7'A	109.0
H2B—C2—H2C	109.5	N2'—C7'—H7'B	109.0
C4—C3—N1	114.3 (7)	C8'—C7'—H7'B	109.0
C4—C3—H3A	108.7	H7'A—C7'—H7'B	107.8
N1—C3—H3A	108.7	C7'—C8'—H8'A	109.5
C4—C3—H3B	108.7	C7'—C8'—H8'B	109.5
N1—C3—H3B	108.7	H8'A—C8'—H8'B	109.5
H3A—C3—H3B	107.6	C7'—C8'—H8'C	109.5
C3—C4—H4A	109.5	H8'A—C8'—H8'C	109.5
C3—C4—H4B	109.5	H8'B—C8'—H8'C	109.5
H4A—C4—H4B	109.5	N2'—C9'—C10'	114.5 (7)
C3—C4—H4C	109.5	N2'—C9'—H9'A	108.6
H4A—C4—H4C	109.5	C10'—C9'—H9'A	108.6
H4B—C4—H4C	109.5	N2'—C9'—H9'B	108.6
N1—C5—C6	114.4 (7)	C10'—C9'—H9'B	108.6
N1—C5—H5A	108.7	H9'A—C9'—H9'B	107.6
C6—C5—H5A	108.7	C9'—C10'—H10D	109.5
N1—C5—H5B	108.7	C9'—C10'—H10E	109.5
C6—C5—H5B	108.7	H10D—C10'—H10E	109.5
H5A—C5—H5B	107.6	C9'—C10'—H10F	109.5
C5—C6—H6A	109.5	H10D—C10'—H10F	109.5
C5—C6—H6B	109.5	H10E—C10'—H10F	109.5
H6A—C6—H6B	109.5	C12'—C11'—N2'	110.1 (7)
C5—C6—H6C	109.5	C12'—C11'—H11C	109.6
H6A—C6—H6C	109.5	N2'—C11'—H11C	109.6
H6B—C6—H6C	109.5	C12'—C11'—H11D	109.6
C8—C7—N2	112.6 (7)	N2'—C11'—H11D	109.6
C8—C7—H7A	109.1	H11C—C11'—H11D	108.1
N2—C7—H7A	109.1	C11'—C12'—H12D	109.5
C8—C7—H7B	109.1	C11'—C12'—H12E	109.5
N2—C7—H7B	109.1	H12D—C12'—H12E	109.5
H7A—C7—H7B	107.8	C11'—C12'—H12F	109.5
C7—C8—H8A	109.5	H12D—C12'—H12F	109.5
C7—C8—H8B	109.5	H12E—C12'—H12F	109.5
H8A—C8—H8B	109.5	N4—C13—N3	118.5 (4)
C7—C8—H8C	109.5	N4—C13—S1	122.8 (3)
H8A—C8—H8C	109.5	N3—C13—S1	118.7 (3)
H8B—C8—H8C	109.5	N3—C14—N5	114.4 (3)
N2—C9—C10	112.6 (7)	N3—C14—S2	122.6 (3)
N2—C9—H9A	109.1	N5—C14—S2	123.0 (3)
C10—C9—H9A	109.1	N4—C15—N5	119.5 (3)
N2—C9—H9B	109.1	N4—C15—S3	123.2 (3)
C10—C9—H9B	109.1	N5—C15—S3	117.3 (3)
H9A—C9—H9B	107.8	N7—C16—N6	119.0 (3)
C9—C10—H10A	109.5	N7—C16—S5	123.8 (3)
C9—C10—H10B	109.5	N6—C16—S5	117.2 (3)
H10A—C10—H10B	109.5	N7—C17—N8	119.6 (3)
C9—C10—H10C	109.5	N7—C17—S6	121.8 (3)
H10A—C10—H10C	109.5	N8—C17—S6	118.6 (3)
H10B—C10—H10C	109.5	N8—C18—N6	113.4 (3)
C12—C11—N2	113.1 (7)	N8—C18—S4	121.9 (3)
C12—C11—H11A	109.0	N6—C18—S4	124.7 (3)
			
C5—N1—C1—C2	179.5 (10)	C15—N4—C13—N3	−2.5 (5)
C3—N1—C1—C2	−51.2 (14)	C15—N4—C13—S1	177.1 (3)
C5—N1—C3—C4	−47.4 (19)	C14—N3—C13—N4	5.6 (6)
C1—N1—C3—C4	−175.2 (16)	C14—N3—C13—S1	−174.1 (3)
C3—N1—C5—C6	−58.1 (17)	C13—N3—C14—N5	−4.0 (6)
C1—N1—C5—C6	68.3 (15)	C13—N3—C14—S2	174.7 (3)
C9—N2—C7—C8	−53.0 (15)	C15—N5—C14—N3	−0.2 (6)
C11—N2—C7—C8	179.2 (12)	C15—N5—C14—S2	−179.0 (3)
C11—N2—C9—C10	65.1 (13)	C13—N4—C15—N5	−1.4 (6)
C7—N2—C9—C10	−62.9 (13)	C13—N4—C15—S3	−179.3 (3)
C9—N2—C11—C12	161.5 (13)	C14—N5—C15—N4	2.9 (6)
C7—N2—C11—C12	−70.5 (15)	C14—N5—C15—S3	−179.1 (3)
C5'—N1'—C1'—C2'	56.9 (13)	C17—N7—C16—N6	1.6 (6)
C3'—N1'—C1'—C2'	−177.0 (12)	C17—N7—C16—S5	−176.9 (3)
C5'—N1'—C3'—C4'	−63.6 (18)	C18—N6—C16—N7	−0.3 (6)
C1'—N1'—C3'—C4'	170.6 (16)	C18—N6—C16—S5	178.3 (3)
C1'—N1'—C5'—C6'	72.4 (15)	C16—N7—C17—N8	−2.9 (6)
C3'—N1'—C5'—C6'	−49.3 (16)	C16—N7—C17—S6	177.7 (3)
C9'—N2'—C7'—C8'	68.6 (15)	C18—N8—C17—N7	3.2 (6)
C11'—N2'—C7'—C8'	−61.2 (15)	C18—N8—C17—S6	−177.4 (3)
C7'—N2'—C9'—C10'	46.7 (15)	C17—N8—C18—N6	−1.9 (6)
C11'—N2'—C9'—C10'	178.3 (12)	C17—N8—C18—S4	177.3 (3)
C9'—N2'—C11'—C12'	58.6 (14)	C16—N6—C18—N8	0.4 (5)
C7'—N2'—C11'—C12'	−169.8 (11)	C16—N6—C18—S4	−178.7 (3)

### Hydrogen-bond geometry (Å, °)

**Table:**

*D*—H···*A*	*D*—H	H···*A*	*D*···*A*	*D*—H···*A*
N1—H1···N4	0.88	2.03	2.89 (1)	163
N1'—H1'···N4	0.88	2.08	2.95 (1)	166
N2—H2···N7	0.88	2.02	2.88 (1)	165
N2'—H2'···N7	0.88	2.04	2.89 (1)	163
N3—H3···S4	0.88 (1)	2.38 (1)	3.248 (3)	169 (4)
N5—H5···S2^i^	0.88 (1)	2.44 (1)	3.319 (4)	171 (3)
N6—H6···S1	0.87 (1)	2.58 (1)	3.446 (3)	170 (3)
N8—H8···S6^ii^	0.88 (1)	2.47 (2)	3.326 (3)	164 (4)

Symmetry codes: (i) −*x*+1, −*y*, −*z*+1; (ii) −*x*, −*y*+1, −*z*+1.
